# Pedestrians’ psychological preferences for urban street lighting with different color temperatures

**DOI:** 10.3389/fpsyg.2022.971700

**Published:** 2022-09-30

**Authors:** Xinyi Hao, Xin Zhang, Jiangtao Du, Meichen Wang, Yalan Zhang

**Affiliations:** ^1^School of Architecture, Tsinghua University, Beijing, China; ^2^School of Architecture, University of Liverpool, Liverpool, Merseyside, United Kingdom; ^3^Graduate School of Architecture, Planning and Preservation, Columbia University, New York City, NY, United States; ^4^A. Alfred Taubman College of Architecture and Urban Planning, University of Michigan, Ann Arbor, MI, United States

**Keywords:** urban street lighting, CCT, video evaluation, pedestrian’s perception, psychological preferences, white LED

## Abstract

White LEDs, which have been widely used in the urban street lighting, are increasingly applied to replace traditional HPS lamps with a lower CCT (correlated color temperature). Generally, studies on the CCT of street lighting focus on providing safe functional lighting for vehicle drivers. However, it is still unknown how the street light color can affect pedestrians’ perception and preferences with respect to lighting levels and ambient temperature.

In this study, a wide range of CCTs (1,600–5,400 K) was measured for urban street lighting in Beijing, China, for example. And the transition from traditional HPS lamps to LEDs lacks a reference street lighting standard for CCT. The study aims to conduct a cross-sensory test to evaluate urban street lighting with multiple combinations of CCT values and illuminance levels according to pedestrians’ visual perception and psychological preferences.

A total of 18 night street lighting scenes with six CCT values and three illuminance levels were first selected in Beijing city, and then HDR videos of these scenes were taken from the view of pedestrians to conduct psychological experiments in an indoor environment with three ambient temperatures. A total of 77 university students (24 males) were invited to assess videos of the 18 lighting scenes in terms of seven factors, such as lighting brightness, color temperature sensation, light color preference, sense of safety, recognition, comfort, and overall preference. Several key findings were achieved as follows. (1) The CCT of urban street lighting can have significant effects on the visual psychological perceptions of participants. (2) There was a significant interaction between CCT, illuminance, and ambient temperature on the visual psychological performances of participants. (3) The higher ambient temperature will deliver the higher level of overall preference for the street lighting with medium and high CCT, and the perception of warmer light color. (4) There was a strong correlation found between participants’ light color preference, comfort, and overall preferences.

## Introduction

With the innovation of LED technology, white LEDs have been widely used in urban street lighting and are gradually replacing traditional low CCT HPS lamps due to their high luminous efficiency and visual acuity (Nardelli et al., 2017). However, at the same time, the widespread use of white LEDs has also brought a series of problems, including uncomfortable visual and psychological perceptions and nighttime rhythmic effects caused by a high proportion of short-wave radiation. As an important carrier for the night life of citizens, the street nightscape should not only provide lighting to guarantee function and safety, but also create a good atmosphere to support the all-weather function of streets in the city (Rong and Zhou, 2021). In recent years, research on street lighting has focused on the design of LED light sources and luminaires to respond to the needs of street lighting at different times and with different characteristics (Curran and Keeney, 2006; Zou, 2010).

In earlier LED street lighting applications, the luminous efficiency of 5,500–6,500 K CCT light sources was much higher than that of neutral light sources around 3,500 K, and thus it was widely adopted. However, with the improvement of LED technology, the difference in luminous efficiency of white LEDs in different CCT zones is gradually being reduced, and the difference in luminous efficiency between warm white light sources of 3,000–3,500 K and cool white light sources of 6,000–6,500 K is less than 6% (Feng and Lu, 2016). The contradiction between luminous efficacy and CCT is no longer the main issue, and the harmony between CCT and the street environment becomes the focus of attention (Feng and Lu, 2016). One of the most recent research hotspots is determining what CCT range is appropriate for urban street lighting.

Established studies have shown that using participants’ preferred lighting can generate positive emotions, increase satisfaction or have a healing effect (Newsham and Veitch, 2001; Newsham et al., 2004; Veitch et al., 2008). Low CCT and low illuminance lighting are more emotionally demanding, making people feel emotionally relaxed and at ease (Hao et al., 2017), while high CCT and high illuminance lighting make participants feel awake and focused, and are conducive to increasing the excitability and attention level of the brain when performing visual tasks (Katsuura et al., 2005; Kim et al., 2017). However, high CCT can also increase visual fatigue and brain fatigue. These findings are from laboratory and office conditions, and studies relevant to real-life situations are still needed to determine how CCT influences people’s psychological perceptions in urban street scenarios.

Spectral power distribution (SPD) and lighting levels of street lighting affect drivers (He et al., 1997; Bullough and Rea, 2000; Akashi et al., 2007; Fotios et al., 2017) and pedestrians (Fotios and Cheal, 2009, 2012; Uttley et al., 2016) in terms of visual performance. Street lighting is in the mesopic visual range, where the spectral luminous efficiency function of the human eye changes, and using visual efficacy to assess light efficiency while driving is more directly practical than optical concepts such as visual brightness (Hurden, 2002). It has been found that when the background luminance is reduced, the human eye’s sensitivity to the spectrum is shifted toward the short-wave direction, and the detection of long-wave visual targets is relatively poor (Lin et al., 2006). In hazy weather with poor penetration of high CCT lighting, it is recommended that the street lighting CCT be in the range of 2,800–4,200 K (Feng and Lu, 2016). The best visual efficacy of 3,500 K CCT can be obtained through actual measurements and surveys (Zhang et al., 2013). Using a light source with a larger color gamut can enhance the color contrast between the target and the background, thus improving the visual efficacy under street lighting conditions (Yang and Wei, 2020). From the pedestrian perspective, identification and intention recognition are important night visual tasks (Fotios and Yang, 2013). Field studies have concluded that MH streetlights (2,726 K) are more likely to achieve better facial recognition than LED streetlights (5,298 K) and HPS lamps (1,930 K; Lin and Fotios, 2013). It was found that for pedestrian paths on campus, lighting CCT of approximately 3,000 K had higher recognition (Yuan et al., 2021b). However, studies on the visual efficacy of street lighting are oriented towards the driver’s perspective, and studies on sidewalk lighting are conducted on stand-alone pedestrian systems with dedicated luminaires. Studies on sidewalk lighting in common Chinese situations, which is indirectly provided by functional street lighting, are lacking.

CCT of light affects the subjective feelings of safety and psychological preferences of motorways and sideways. For example, CCTs that are psychologically considered most suitable for motorway lighting include 4,000 K (Beccali et al., 2019), 4,100–4,300 K (Beckwith et al., 2010), while street lights with too high a CCT (Luo et al., 2013) or 5,500–6,000 K (Beckwith et al., 2010) are uncomfortable. Lighting is strongly correlated with the perception of safety on walking paths (Fotios and Goodman, 2012; Fotios and Unwin, 2013; Fotios et al., 2015), and the CCTs that are psychologically considered most suitable for walking paths include 3,000 K (Jin et al., 2015; Davidovic et al., 2018; Yuan et al., 2021b), 3,000 K/5 lx or 3,500 K/50 lx (Petrulis et al., 2017), and 3,800 K (Yuan et al., 2021a). Although the above studies did not form a unified conclusion, it can still be summarized that the appropriate CCT of motorways is higher than that of sideways, and the difference between the two should be paid attention to due to the large number of cases in China where sideway lighting is provided indirectly by motorway lighting.

The current Chinese Urban Road Lighting Design Standard (Ministry of Housing and Urban-Rural Development of the People’s of China, 2015) for street lighting states that the CCT should not be higher than 5,000 K and that it is advisable to give preference to medium/low CCT light sources, otherwise comfort will be affected. The current white LED CCT range has been widened to between 1,700 and 18,000 K (Kokka et al., 2018). In Beijing, for example, the typical CCT intervals of street lighting measured randomly include 1,600–2,200 K, 2200–2,700 K, 2700–3,200 K, 3,600–4,300 K, 4,300–4,900 K, and 4,900–5,400 K. Usually, research on the CCT of street lighting focuses on functional lighting based on the driver’s perspective, while the preferences of pedestrians and whether the preferences are related to illuminance and ambient temperature need to be further explored, as a supplement to the driver’s perspective research. Also, since most streets have functional lighting that also serves as sidewalk lighting, it can help to better understand pedestrian preferences for urban functional lighting and provide data support for sidewalk lighting.

Current white LED technology already enables reliable CCT adjustment. If the psychological preference of pedestrians or passengers for street lighting is dynamic, e.g., related to outdoor temperature and noise, the setting can be adjusted according to seasonal climate, outdoor environment, and roadway type characteristics. This study conducts experiments across sensory channels to focus on the way ambient temperature affects CCT preferences of street lighting.

When it is difficult to meet the requirement of conducting evaluation studies in real scenarios, the method of image evaluation can be used to present real situations through pictures or dynamic videos. Subjective quantitative evaluation is performed by participants in the laboratory under the premise of ensuring the consistency of the optical properties. Studies have shown that image reproduction of real scenes can be used instead of field evaluation (Manav, 2013), and dynamic video has also been used as a research tool for image evaluation in studies of environmental psychology and urban landscapes, capable of reflecting the dynamic properties of urban environmental horizons (Ode et al., 2008). High dynamic range (HDR) image technology is able to perform image simulation of original scenes based on multi-exposure dynamic range (Inanici, 2006; Wang, 2011), which has some advantages in subjective evaluation and has great potential for street lighting measurement with high luminance contrast. Therefore, this study uses HDR video evaluation to study street lighting to solve the problems of many disturbing factors and the uncontrollable temperature of field experiments, and also to achieve a greater degree of restoration of real scenes.

This study investigated typical streets in Beijing, measured CCT and illuminance, categorized 18 lighting combinations with six CCT values and three illuminance zones, and captured HDR videos of pedestrian view. Under three indoor temperatures (19°C, 24°C, and 29°C), 77 participants were invited to view the 18 videos indoors and complete a Likert scale to obtain their preference evaluation of different street lighting combinations under different ambient temperatures. Starting from the psychological preference of pedestrians, we provide human factors data support for the improvement of street lighting standards beyond the perspective of driving safety, and provide suggestions for the design and dynamic regulation of street lighting in different climate zones through the exploration of cross-sensory channels.

## Materials and Methods

### Experimental site and equipment

**Figure 1:**
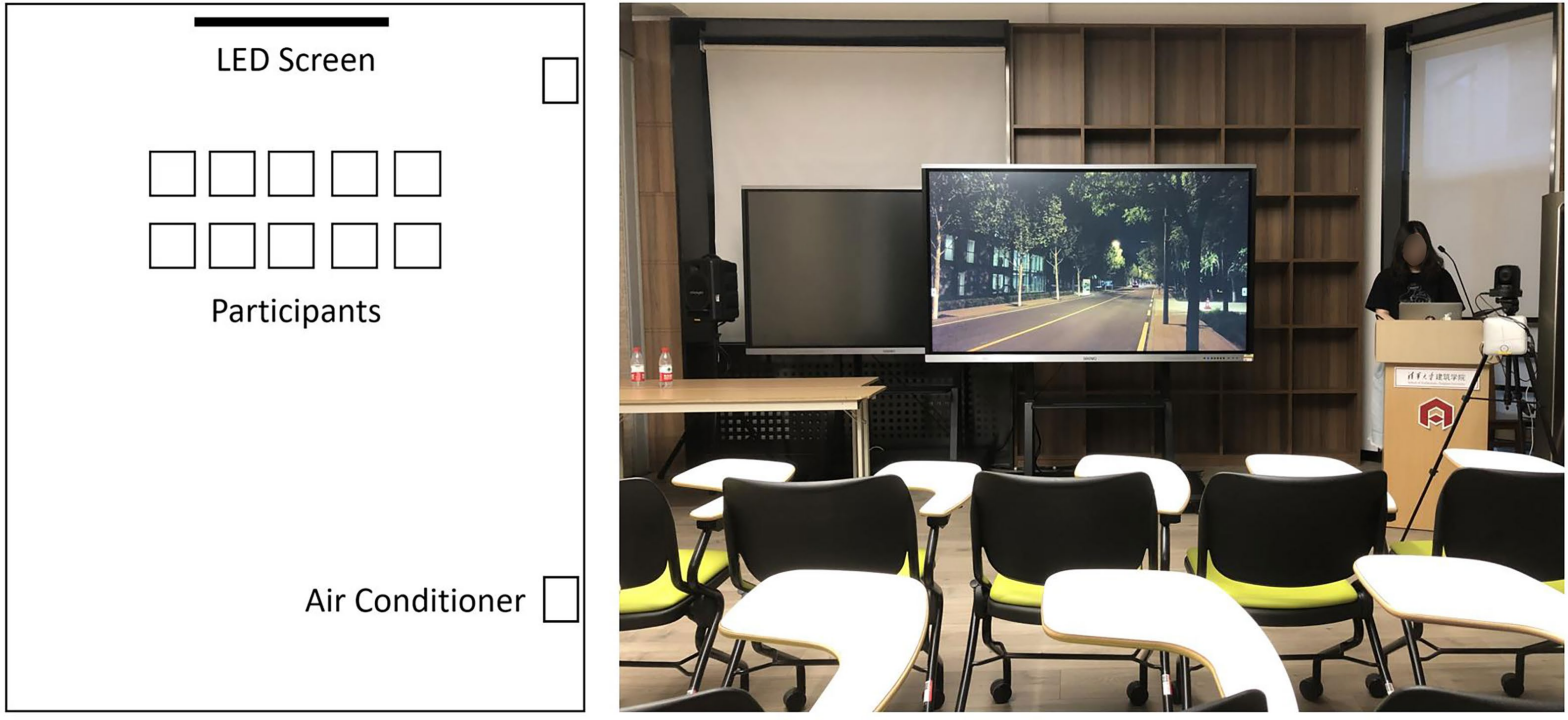
Experimental scene of street lighting evaluation (indoor lighting turned off during the experiment).

A classroom was used as the evaluation laboratory, with a length of 12 m, a width of 6.3 m, and a net height of 3.8 m (Figure 1). The length of the LED display screen supporting 4 K resolution was 1.9 m, and the top and bottom edges were 2 m and 0.9 m from the floor, respectively. A total of 10 participants were seated in two rows, five in each row, with 0.3 m between their shoulders, and the front row participants were 2.5 m away from the screen, so that their sight lines were not blocked and the difference in viewpoint was small. All lights inside and outside the classroom were turned off during the experiment. The curtains of the south window were drawn. The room was slightly illuminated by the light transmitted from the adjacent building on the south side, and no reflections were formed on the ceiling and walls when the display screened videos. The room temperature was adjusted to 19°C, 24°C, and 29°C using air conditioners, representing cool, neutral, and hot ambient temperatures, respectively. Room temperature was measured using a thermo-hygrometer, and there was no significant temperature difference between the areas where the participants were located. The relative humidity in the room was all controlled at about 35–40%.

### Pre-experiment

**Figure 2:**
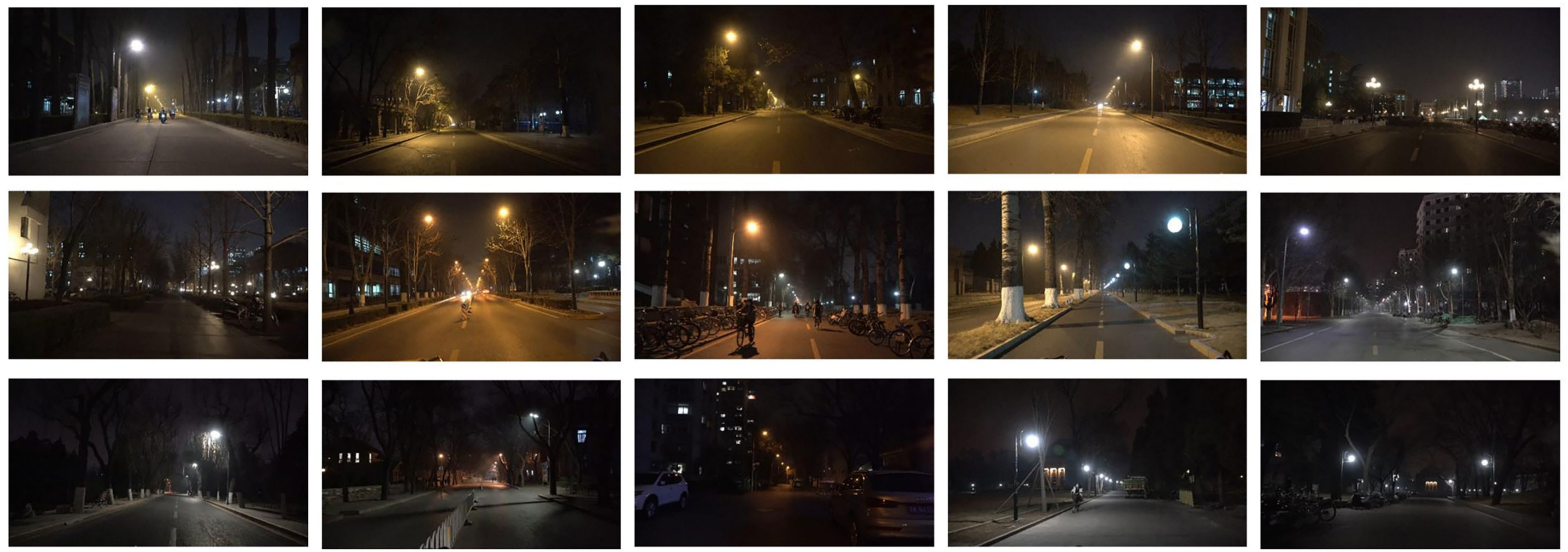
Part of the street scenes of the campus pre-experiment.

In order to verify the effectiveness of image evaluation and video evaluation, a pre-experiment was conducted with the street lighting scene in Tsinghua University campus as an example (Figure 2). Photographs and videos of 14 locations were captured at 20–21 PM using a motion camera (DJI OSMO POCKET), and evaluation questionnaires were completed by 20 participants in the lab, and the same participants were invited to the field for evaluation at the same time the next night. The evaluation factors included lighting brightness, color temperature sensation, light color preference, and overall preference. Paired-samples T-tests were conducted for these four factors, and statistically significant differences were obtained for both photo and field evaluations (*p* < 0.05), while the differences between video and field evaluations were not statistically significant (*p* > 0.05). That is, the video evaluation was closer to the on-site evaluation results than the photo, so the video was selected as the experimental evaluation material.

### Lighting scenes for evaluation

**Figure 3:**
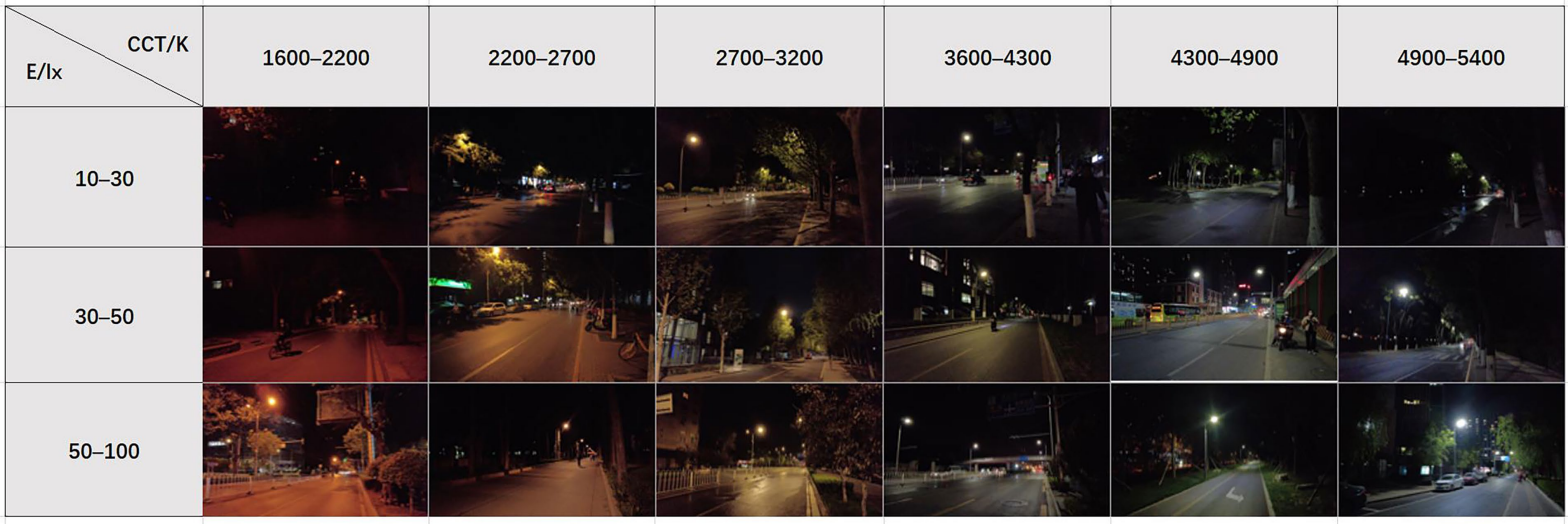
Images of streets with different CCT and illuminance combinations (shot in April to May 2021, 19–21 PM).

Field study on different streets in Beijing, measuring CCT and illuminance, categorized into six CCT values and three illuminance levels, for a total of 18 combinations of actual street sections (Figure 3). In the 18 typical road lighting sections, after holding a motion camera (DJI OSMO POCKET) to human eye height and adjusting the white balance on site until there was no difference between human eye perception and the camera display, 4 K HDR videos were taken at an angle of 30 degrees from the sidewalk near the motorway to the opposite side of the field of view, moving at an even pace to simulate the street scenes seen on foot.

All videos were adjusted to the view of walking on the right side of the street, and clips with a walking range of approximately the distance between streetlights were taken in each video, using the clips with less obscured vehicles and street signs in the image as samples. Each video’s length was about 15–30 s, as was the experimental evaluation material.

### Participants

From May 24 to 29, 2021, from 19:30 to 22:00 every night, 77 students (24 men and 53 women, 20–22 years old) in their third year of undergraduate studies at the School of Architecture, Tsinghua University, participated in the experiment. The participants had a relatively in-depth understanding of the concepts of the built environment and lighting. Each participant evaluated 18 videos of street scenes at three room temperatures. Prior to each experiment, participants were informed of the room temperature for the day and dressed accordingly with appropriate clothing. They were divided into groups of 10, with myopic students wearing glasses with normal corrected vision and no participants with color vision weakness or color vision abnormalities.

### Experimental procedure

The videos were tuned by image processing software and measured using a spectral illuminance meter to ensure that the illuminance and CCT of the eyes of the participants would be approximately the same as the lighting at the pedestrian location in the real situation corresponding to the video presented. The processed videos were randomly sorted and stored in groups, and five sets of experiments at the same room temperature were conducted each night. A total of 10 sets of experiments were completed at each room temperature, all with different random sorting.

The laboratory was prearranged, and the air conditioning temperature was adjusted until the participant area reached the preset value, and the indoor humidity was recorded. Each group of 10 subjects entered the classroom and underwent a 5-min dark adaptation and temperature adaptation while the experimenter explained the experiment content and rules. The display screen looped the first video, and participants could fill in the questionnaire at any time during the viewing process. The questionnaire used a 7-level Likert scale to evaluate lighting brightness, color temperature sensation, light color preference, recognition, sense of safety, comfort, and overall preference. After everyone filled out the evaluation of the first video, they moved on to the second one, thus completing the 18 videos and the evaluation. The participants were given sufficient observation and feeling time (the observation time of each video clip in the actual experiment is about 2 min), and the length of the experiment was about 40 min for each group. The participants submitted the questionnaire and made sure that it was filled out correctly before leaving. The next group entered the classroom for the second set of experiments, and they completed the five sets of experiments each night in turn.

### Data analysis

The study used a repeated-measures experimental design, and the independent variables included three within-subjects factors (CCT, illuminance, and experimental temperature). The dependent variables included 7 semantic difference scales: lighting brightness (insufficient/sufficient), color temperature sensation (cold/warm), light color preference (dislike/like), recognition (cannot be accurately recognized/can be accurately recognized), sense of safety (danger/safety), comfort (discomfort/comfort), and overall preference (dislike/preference). Each factor was evaluated using a 7-point Likert scale.

IBM SPSS Statistics was used for data analysis. Firstly, descriptive statistics were performed on the seven evaluation factors to obtain the basic information of the evaluation results. Then correlation analysis and factor analysis were performed on the evaluation factors to explore the correlation between them and extract the principal components. Next, a three-factor repeated-measures ANOVA was conducted on CCT, illuminance, and experimental temperature to explore whether there was an interaction between the three and whether there was an effect on the seven evaluation factors. The conditions to be satisfied were (1) the data in each group basically conformed to normal distribution by the Q-Q plot test; (2) there were no extreme outliers in each group judged by box plots; and (3) the variance covariance matrix of the dependent variables was equal (*p* > 0.05) for the interaction term CCT* illuminance* experimental temperature by Mauchly’s spherical hypothesis test; and (4) if they were not equal (*p* < 0.05), the Grennhouse-Geisser or Huynh-Feldt coefficients were selected for epsilon correction. If there was an interaction between the three factors, then (1) continue to test whether there was a simple two-factor interaction; (2) if there was a simple two-factor interaction, continue to test whether there was a simple effect; and (3) if so, continue to test whether a simple two-by-two comparison was significant.

## Results

### Factor analysis of dependent variables

Before factor analysis, correlations between dependent variables were first studied. The Likert scale results used in this study were ordered categorical variables, all of which were analyzed using Kendall’s tau-b correlations, with correlation coefficients less than 0.4 being weak correlations, 0.4–0.7 being moderately strong correlations, and greater than 0.7 being strong correlations.

**Figure 4:**
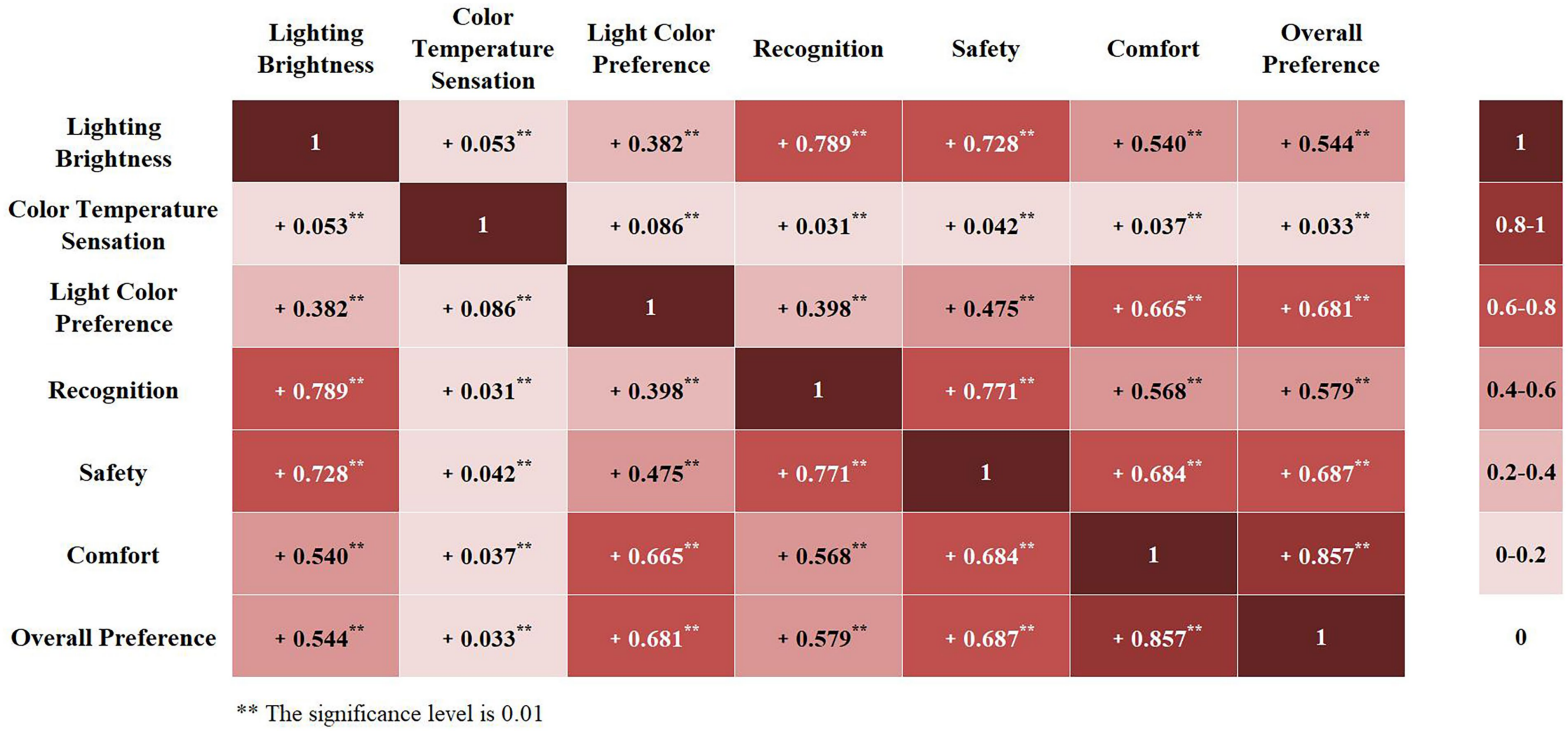
Heat map of the correlation coefficient matrix between dependent variables.

A two-by-two correlation analysis was performed on the seven evaluation factors at all experimental temperatures to establish a correlation coefficient matrix (Figure 4). The results were as follows. (1) All independent variables are positively correlated. (2) A weak correlation between lighting brightness and color temperature sensation, light color preference; between color temperature sensation and light color preference, recognition, safety, comfort, and overall preference. (2) A moderately strong correlation between brightness and comfort, overall preference; between light color preference and recognition, safety, comfort, and overall preference; between recognition and comfort, overall preference; and between safety and comfort, overall preference. (3) A strong correlation between brightness and recognition, safety; between recognition and safety; and between comfort and overall preference. Among them, the factors with the highest degree of correlation with light color preference and overall preference are all comfort, and there is also a strong correlation between light color preference and overall preference.

For the six dependent variables of lighting brightness, color temperature sensation, light color preference, recognition, safety, and comfort, the principal components were extracted (Table 1). The data structure is reasonable (KMO test coefficient is 0.820, and *p* < 0.001 for Bartlett’s test results), and factor analysis can be performed.

**Table 1:** Rotated component matrix.

	Component 1	Component 2
Safety	0.939	
Recognition	0.909	
Lighting brightness	0.895	
Comfort	0.894	
Light color preference	0.743	
Color temperature sensation		0.999

The results of factor analysis suggest that the eigenvalues of the top 2 principal components are greater than or equal to 1, explaining 64.382, and 16.664% of the total data variance, respectively, and the correlation between the two factors is low (correlation coefficient less than 0.1). Therefore, the top 2 principal components were finally extracted, and the extracted principal components explained 81.046% of the data variance cumulatively. From the rotated component matrix (Table 1), it can be obtained that principal component 1 has a high correlation with lighting brightness, light color preference, recognition, safety, and comfort, which can be referred to as the participant’s psychological perception; principal component 2 has a high correlation with color temperature sensation, which can be referred to as the participant’s warm and cold perception.

### Effects of lighting and temperature on overall preference

**Figure 5:**
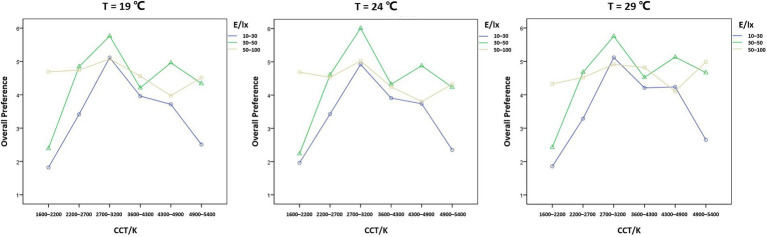
Interaction trend of CCT, illuminance, and experimental temperature on the overall preference score.

A three-factor repeated-measures ANOVA was used to determine the effects of CCT, illuminance, and experimental temperature on the evaluation of overall preference (Figure 5).

The interaction between CCT, illuminance, and experimental temperature had a statistically significant effect on the overall preference score, *F* = 2.203, *p* = 0.003 < 0.05. Therefore, a simple two-factor interaction test was performed.

The interaction of CCT and illuminance was chosen to be verified at different levels of experimental temperature. When the experimental temperature was 19°C, *F* = 47.709, *p* = 0.000 < 0.05; when the experimental temperature was 24°C, *F* = 45.592, *p* = 0.000 < 0.05; when the experimental temperature was 29°C, *F* = 51.966, *p* = 0.000 < 0.05. To sum up, the interaction of CCT and illuminance was statistically significant at all three experimental temperatures.

The effect of CCT on overall preference scores was statistically significant for all nine combinations of levels with experimental temperatures of 19, 24, and 29°C and illuminance levels of 10–30, 30–50, and 50–100 lx, respectively, and all had a simple effect, *p* = 0.000 < 0.001. Pairwise comparisons revealed that most of the differences between two of the six CCT levels in the nine cases were statistically significant (*p* < 0.001).

The ranking of the ratings for overall preference at different temperatures was obtained after participants watched the videos of 18 CCT and illuminance combinations at three experimental temperatures. At the neutral temperature of 24°C, the top three for overall lighting preferences were all 2,700–3,200 K: CCT3E2 (30–50 lx), CCT3E3 (50–100 lx), and CCT3E1 (10–30 lx). At a cooler temperature of 19°C, the top three for overall preferences were still 2,700–3,200 K, respectively: CCT3E2 (30–50 lx), CCT3E1 (10–30 lx), and CCT3E3 (50–100 lx), and the overall preference score for low and medium CCT lighting was higher than 24°C. At a warmer temperature of 29°C, the top three for overall preferences were: CCT3E2 (2,700–3,200 K, 30–50 lx), CCT5E2 (4,300–4,900 K, 30–50 lx), and CCT3E1 (2,700–3,200 K, 10–30 lx), and participants’ overall preference ratings for medium and high CCT lighting were higher than at 24°C. The participants’ overall preference scores for roadway lighting were identical in the last three: CCT1E1 (1,600–2,200 K, 10–30 lx), CCT1E2 (1,600–2,200 K, 30–50 lx), and CCT6E1 (4,900–5,400 K, 10–30 lx). That is lighting conditions with low CCT and low illuminance, or high CCT and low illuminance.

### Effects of lighting and temperature on psychological perception

The interaction between CCT, illuminance, and experimental temperature had a statistically significant effect on recognition and safety ratings. For recognition, *F* = 2.799, *p* = 0.000 < 0.05. For safety, *F* = 2.535, *p* = 0.001 < 0.05. Therefore, a simple two-factor interaction test was conducted to obtain a statistically significant effect of the interaction between CCT and illuminance on both recognition and safety for all three temperatures.

The effects of CCT on recognition and safety scores were statistically significant at nine combinations of levels with experimental temperatures of 19, 24, and 29°C and illuminance levels of 10–30, 30–50, and 50–100 lx, respectively, all with simple effects, *p* = 0.000 < 0.001. Pairwise comparisons revealed that most of the differences between two of the six CCT levels in the nine cases were statistically significant (*p* < 0.001).

When the illuminance is 10–30 lx, the optimal CCT for both pedestrian recognition and safety is 2,700–3,200 K. When the illuminance is 30–50 lx, the optimal CCT for pedestrian recognition is 4,300–4,900 K, and for pedestrian safety is 2,700–3,200 K. When the illuminance is 50–100 lx, the optimal CCT for both pedestrian recognition and safety is 1,600–2,200 K. The results show that the optimal CCT for both pedestrian recognition and safety is 2,700–3,200 K overall (Table 2), which is lower than the applicable lighting for motorway safety and visual efficacy compared with the existing studies, but basically in line with the pedestrian sideway lighting safety perception and recognition requirements.

**Table 2:** The optimal CCT and mean values of participants’ scores correspond to recognition and safety at different temperatures.

Psychological perception	E/lx	19°C		24°C		29°C	
		Optimal CCT/K	Mean	Optimal CCT/K	Mean	Optimal CCT/K	Mean
Recognition	10–30	2,700–3,200	4.74	2,700–3,200	4.66	2,700–3,200	4.88
	30–50	4,300–4,900	6.32	4,300–4,900	6.21	2,700–3,200	6.14
	50–100	1,600–2,200	6.00	1,600–2,200	6.17	1,600–2,200	5.84
Safety	10–30	2,700–3,200	4.84	2,700–3,200	4.64	2,700–3,200	5.08
	30–50	4,300–4,900	6.16	2,700–3,200	6.05	2,700–3,200	6.09
	50–100	1,600–2,200	5.84	1,600–2,200	5.95	1,600–2,200	5.77

### Effects of lighting and temperature on cold and warm perception

**Figure 6:**
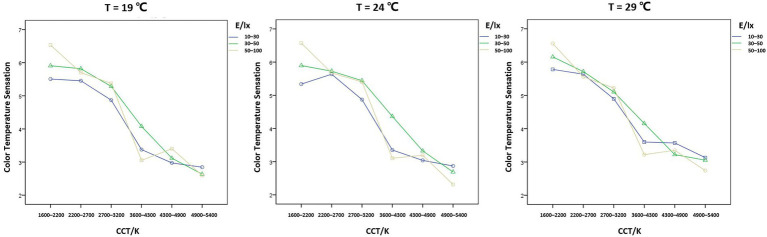
Interaction trend of CCT, illuminance, and experimental temperature on the color temperature sensation score.

The interaction between CCT, illuminance, and experimental temperature on color temperature sensation had a statistically significant effect on the ratings of color temperature sensation (Figure 6), *F* = 38.264, *p* = 0.005 < 0.05. Therefore, a simple two-factor interaction test was performed.

The interaction between CCT and illuminance was chosen to be verified at different levels of the experimental temperature. When the experimental temperature was 19°C, *F* = 17.139, *p* = 0.000 < 0.05; when the experimental temperature was 24°C, *F* = 21.507, *p* = 0.000 < 0.05; when the experimental temperature was 29°C, *F* = 14.903, *p* = 0.000 < 0.05. That is, the interaction between CCT and illuminance was statistically significant at all three experimental temperatures. At the same temperature, low illuminance of 10–30 lx made for a cooler overall feeling, and the opposite was true for medium illuminance of 30–50 lx. This effect was more pronounced at a temperature of 24°C. At the same CCT, higher illuminance conditions made the participants perceive the CCT warmer. For example, at CCT levels of 1,600–2,200 K, 2,700–3,200 K, and 4,300–4,900 K, the participants perceived the CCT warmer at 50–100 lx than at 10–30 lx. Interestingly, at 50–100 lx, the participants all thought that the CCT of 3,600–4,300 K felt cooler than 4,300–4,900 K.

By comparing the group differences between the three temperatures at the six CCT levels, it was determined that the experimental temperature was able to influence the participants’ perception of coldness and warmth. At the experimental temperature of 19°C, the participants perceived the CCT as colder, and this effect was especially seen in the CCT levels of 3,600–4,300 K and 4,300–4,900 K. At the experimental temperature of 29°C, the participants perceived the CCT as warmer, especially in the CCT levels of 1,600–2,200 K, 3,600–4,300 K, 4,300–4,900 K, and 4,900–5,400 K.

## Conclusion

Participants viewed videos of 18 CCT and illuminance combinations at three experimental temperatures to obtain different evaluation factor scores for each scene at different temperatures.

**Figure 7:**
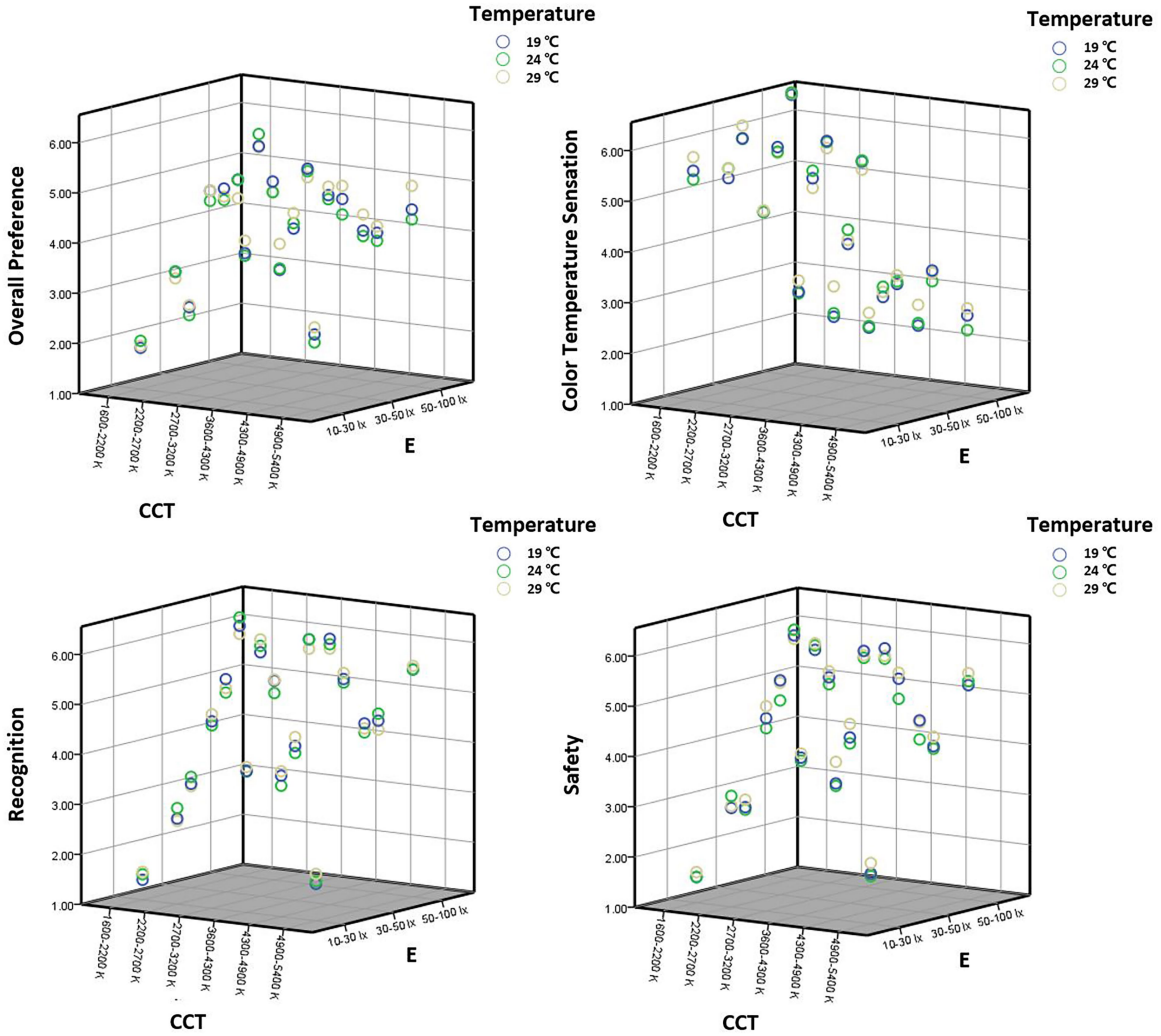
Evaluation scatter plots of different street lighting at three experimental temperatures.

**Figure 8:**
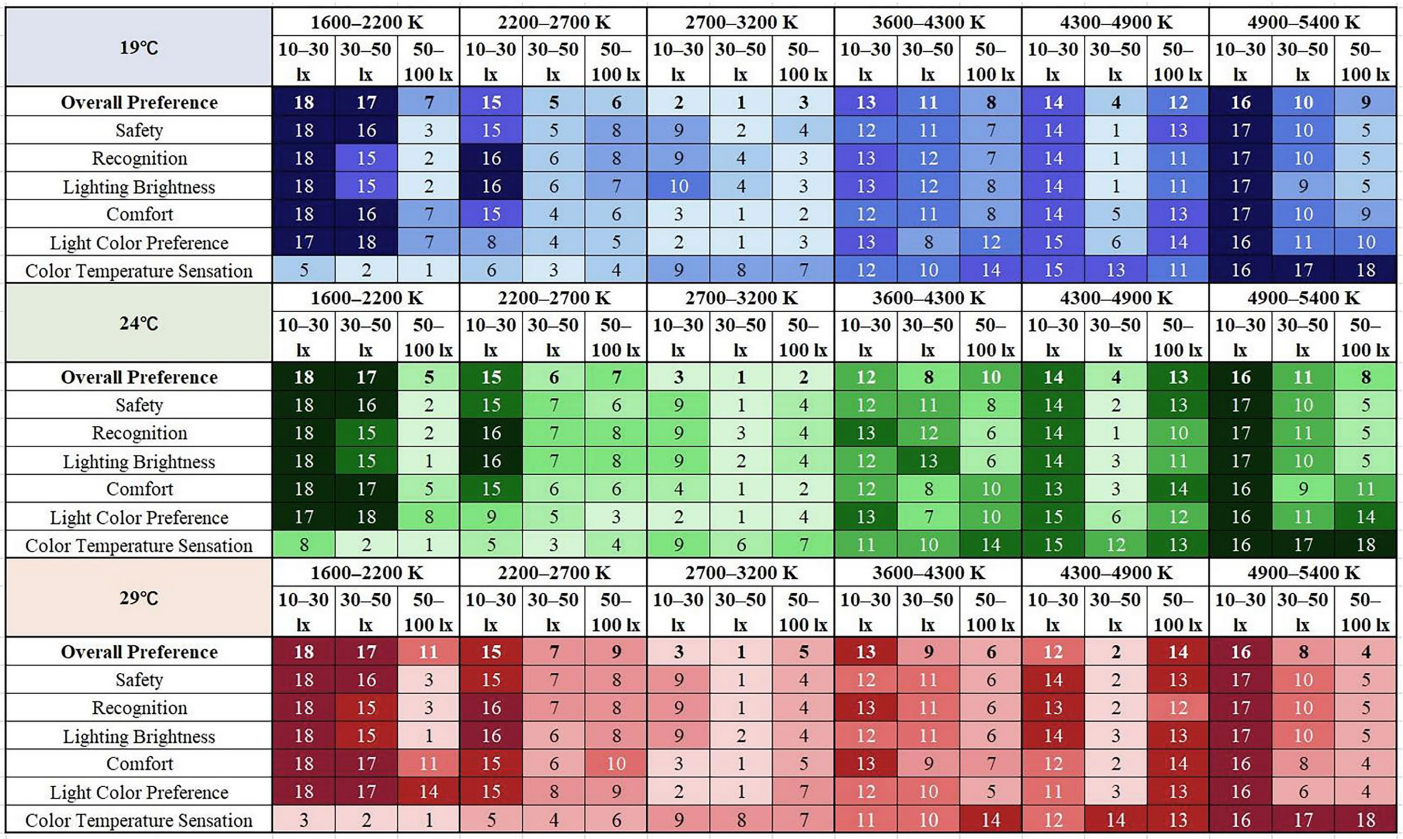
Evaluation rankings of different street lighting under three experimental temperatures.

The overall preference scores and the recognition, safety, comfort, and light color preference scores showed similar trends at different temperatures (Figure 7). According to the Chinese road lighting standard (2015), most of the motorway lighting is below 30 lx, i.e., for the 10–30 lx interval in this study, the optimal CCT range is 2,700–3,200 K, with large differences in preference between different CCT levels. For 30–50 lx, the best CCT range is 2,700–3,200 K, followed by 4,300–4,900 K. For the high illuminance range of 50–100 lx, the best CCT range is 2,700–3,200 K, with little difference in preference between different CCT levels (Figure 8).

Pedestrians’ psychological perception of CCT is not only related to the illuminance level of the street but also to the ambient temperature they are exposed to. The subjective evaluation of participants for different CCT and illuminance level combinations differed under different experimental temperatures. There is a three-factor interaction between temperature, CCT, and illuminance. Specifically, the interaction of CCT and illuminance existed at different experimental temperatures. And on different combinations of experimental temperature and illuminance, CCT had a significant effect on the ratings of lighting perception.

By observing the statistical plots of ratings, it was found that temperature affects participants’ overall preference for street lighting and the warm and cold perceptions of CCT. The higher the temperature, the better the participants’ overall preference for medium and high CCT levels. The higher the temperature, the warmer the participants’ perception of CCT. In the interval of 10–30 lx, which reflects the level of street lighting in China, the overall preference for lighting at 29°C was higher than that of 19°C and 24°C.

## Discussion

According to the model proposed by Rea et al. (2011), for outdoor scene brightness perception, the brightness sensitivity of the human eye increases relatively to the short wavelength spectrum. The overall brightness under 20 lx illuminance conditions (measured 17 lx) for CMH 4,200 K (measured 3,750 K) and MV (measured 4,052 K) is judged to be higher than for CMH 2,800 K (measured 2,583 K). Under the 10–30 lx conditions in this study, the 2,700–4,300 K lighting was overall higher than the 1,600–2,700 K traditional lighting in terms of brightness perception and safety ratings. However, the mean measured illuminance of 2,700–4,300 K lighting was 13.5 lx, which was lower than the mean measured illuminance of 1,600–2,700 K lighting of 19 lx. The model of Rea et al. (2011) helps to understand the results of this study. Combined with the findings of existing studies (Boyce et al., 2000; Rea et al., 2009) that suggest the brightness perception in outdoor environments is related to the sense of safety, it has practical utility for lighting standards.

In the common street lighting situation in the mesopic visual range, the results of this study are low compared with the CCT obtained from the existing studies based on the motorway safety perspective, but are generally consistent with the CCT obtained from the sidewalk safety perspective. The results of this study are lower than those obtained from the existing studies based on the motorway identification degree, but basically consistent with the CCT obtained from the pedestrian recognition degree.

Priority should be given to the visual requirements of drivers, such as identification from a safety standpoint, in the design of urban street lighting. The findings of this study prove that LED motorway lighting is usually high in CCT for the sidewalks that borrow its lighting, which is not the best preference. Lower CCT lighting of 2,700–3,200 K should be appropriately supplemented in the pedestrian area, taking into account the visual preference of pedestrians. Because individual physiological and psychological characteristics may affect light color preference, this study is only valid for people with characteristics such as region and age represented by the test sample.

In addition to the CCT itself, other non-optical factors may affect the overall preference, such as temperature and other environmental physical parameters. In the planning and design of street lighting, cross-sensory factors should also be taken into account, such as the dynamic adjustment of CCT according to the ambient temperature.

With the establishment of the evaluation system for color gamut and color saturation, and the innovation of the convenience of wearable spectral measurement devices, the physiological-psychological effects of spectral power distribution and color rendering performance should be explored more carefully in street lighting research, in addition to the CCT. In this study, a video evaluation method was used to obtain subjective data from the participants. If supplemented with physiological data monitoring methods such as electroencephalogram (EEG), galvanic skin response (GSR), eye tracking, and heart rate, it will improve the assessment of light color preference.

## Data availability statement

The original contributions presented in the study are included in the article/Supplementary material, further inquiries can be directed to the corresponding author.

## Ethics statement

The studies involving human participants were reviewed and approved by Institutional Review Board of Tsinghua University. The patients/participants provided their written informed consent to participate in this study.

## Author contributions

XH participated in the design of the study, carried out the experiment, performed the statistical analysis, and drafted the manuscript. XZ proposed the concept, participated in the design of the study, and helped to draft the manuscript. JD proposed the concept. MW and YZ helped to carry out the experiment. All authors contributed to the article and approved the submitted version.

## Funding

This work was supported by a grant from the Tsinghua-Toyota Joint Research Fund (grant number 20213930037) and the National Natural Science Foundation of China (grant number 52078266) for experimental expenses.

## Conflict of interest

The authors declare that the study was conducted in the absence of any commercial or financial relationships that could be construed as a potential conflict of interest.

## Publisher’s note

All claims expressed in this article are solely those of the authors and do not necessarily represent those of their affiliated organizations, or those of the publisher, the editors and the reviewers. Any product that may be evaluated in this article, or claim that may be made by its manufacturer, is not guaranteed or endorsed by the publisher.
